# Toxoplasma and coxiella infection and psychiatric morbidity: A retrospective cohort analysis

**DOI:** 10.1186/1471-244X-4-32

**Published:** 2004-10-18

**Authors:** Hollie V Thomas, Daniel Rh Thomas, Roland L Salmon, Glyn Lewis, Andy P Smith

**Affiliations:** 1 Department of Psychological Medicine, University of Wales College of Medicine, Heath Park, Cardiff, UK; 2 Communicable Disease Surveillance Centre, National Public Health Service, Wedal Road, Cardiff, UK; 3 Division of Psychiatry, University of Bristol, Cotham Hill, Bristol, UK; 4 Centre for Occupational and Health Psychology, School of Psychology, Cardiff University, Cardiff, UK

## Abstract

**Background:**

It has been suggested that infection with *Toxoplasma gondii *is associated with slow reaction and poor concentration, whilst infection with *Coxiella burnetii *may lead to persistent symptoms of fatigue.

**Methods:**

425 farmers completed the Revised Clinical Interview Schedule (CIS-R) by computer between March and July 1999 to assess psychiatric morbidity. Samples of venous blood had been previously collected and seroprevalence of *T. gondii *and *C. burnetii *was assessed.

**Results:**

45% of the cohort were seropositive for *T. gondii *and 31% were positive for *C. burnetii*. Infection with either agent was not associated with symptoms reflecting clinically relevant levels of concentration difficulties, fatigue, depression, depressive ideas or overall psychiatric morbidity.

**Conclusions:**

We do not provide any evidence that infection with *Toxoplasma gondii *or *Coxiella burnetii *is associated with neuropsychiatric morbidity, in particular with symptoms of poor concentration or fatigue. However, this is a relatively healthy cohort with few individuals reporting neuropsychiatric morbidity and therefore the statistical power to test the study hypotheses is limited.

## Background

It has been suggested that infections with the zoonoses *Toxoplasma gondii *and *Coxiella burnetii *may lead to long term neuropsychiatric morbidity. More specifically, *T. gondii *infection is hypothesised to be associated with slow reaction and poor concentration [1,2] whilst *C. burnetii *infection is reported to be associated with persistent symptoms of fatigue for up to ten years following exposure [3-5]. Exposure to the parasitic protozoon *T. gondii *is common in the UK population as a whole (40% to 50%) whereas exposure to the rickettsia-like *C. burnetii *leading to Q fever is relatively rare in urban populations [6-8].

We have examined data previously collected from a farm-based occupational cohort recruited in three areas of England to test these hypotheses. Furthermore, given the high risk of suicide amongst farmers as an occupational group[9], we also investigated associations between infection with either organism and symptoms of depression or depressive ideas. Statistical associations were examined between measures of lifetime exposure to *T. gondii *and *C. burnetii *and current psychiatric morbidity measured by the Revised Clinical Interview Schedule (CIS-R).

## Methods

### Sample

A representative cohort of 606 farmers, farmworkers and family members has been recruited since 1991 in three areas of England to investigate occupational risk factors for zoonoses[10]. A random sample of farmers was drawn from the Ministry of Agriculture, Fisheries and Food June Agricultural Census lists of agricultural holdings[11], and each farmer could then nominate a further adult on the same farm holding (usually his wife). Seventy-seven per cent of the cohort were still enrolled in May 1998 and of these, 425 (91%) completed the CIS-R by computer between March and July 1999[12].

### Psychiatric morbidity data

The computer-administered version of the CIS-R was used to assess the prevalence of symptoms of neurotic psychopathology in the week prior to interview[13]. The CIS-R is made up of fourteen sections, each covering a particular area of neurotic symptoms. For this study we utilized data from the sections relating to fatigue, concentration difficulties, depression and depressive ideas as assessment of psychiatric outcome. Individual symptoms are regarded as clinically relevant if they have a score of two or more (range zero to four, or five for section on depressive ideas). Summed scores from all fourteen sections range from zero to fifty seven, the overall threshold for clinically significant psychiatric morbidity is twelve. The time taken to complete the questionnaire ranged from ten to thirty minutes, due to the filtering nature of the questions.

As reported previously, the prevalence of clinically relevant levels of each of these neurotic symptoms was relatively low[9]. Fifteen percent of farmers reached the threshold for a clinically relevant level of symptoms of fatigue (n = 62). Approximately 5% of the farmers reported symptoms of either concentration difficulties (n = 22) or depressive ideas (n = 23), and 4% reported symptoms of depression (n = 18). Approximately 6% of farmers reported significant general psychiatric morbidity (n = 25).

### Seroprevalence data

At enrolment, 10 ml of venous blood was taken from all subjects. Samples were screened for *Toxoplasma gondii *at Hereford PHL using the Eiken latex agglutination test (cut off titre 1/32). Seroconverters were confirmed by PHLS Toxoplasma Reference Laboratory at Swansea PHL using the dye test and by IgM ELISA. Serum IgG specific antibody levels for *Coxiella burnetii *phase II antigen were estimated at Bristol Public Health Laboratory (PHL) using an indirect immunofluorescence test. Serum with a reciprocal titre of 32 or more were taken as positive. Seroprevalence data for *T. gondii *and *C. burnetii *were available for 370 and 422 individuals respectively.

### Statistical analysis

All analyses allowed for clustering by farm holding in the survey data (Stata Version 6.0, StataCorp, College Station, TX, USA). Odds ratios (ORs) and 95% confidence intervals (95% CIs) were calculated using logistic regression and were adjusted for sex and age in ten year bands.

## Results

In total, 45% (95% CI 40% to 50%) of the cohort were seropositive for *T. gondii *and 31% (95% CI 26% to 36%) were positive for *C. burnetii*. Forty six individuals were seropostivie for both. Exposure to either infection did not differ significantly between men and women (*T. gondii *seroprevalence 47% vs. 41%; *C. burnetii *seroprevalence 32% vs. 28%). *T. gondii *seroprevalence increased significantly with age (<30 years 9%, 30–39 years 29%, 40–49 years 33%, 50–59 years 54%, 60–69 years 57%, 70+ years 56%; χ^2 ^= 25.8, df = 5, P = 0.0002). *C. burnetii *seroprevalence was not associated with age (<30 years 38%, 30–39 years 28%, 40–49 years 36%, 50–59 years 27%, 60–69 years 29%, 70+ years 36%).

Those seropositive for *T. gondii *were not more likely to report symptoms reflecting clinically relevant levels of fatigue, concentration difficulties, depression, depressive ideas or overall psychiatric morbidity than those who were seronegative (Table 1). Similarly, evidence of infection by *C. burnetii *was not significantly associated with reporting of clinically relevant levels of any of these symptoms. Indeed, although based on very limited numbers, a greater percentage of farmers who were seronegative rather than seropositive for *C. burnetii *reported psychiatric symptoms. Furthermore, neither infection was associated with an increased risk of any studied psychiatric outcome after adjusting for age and sex (Table 2).

**Table 1:** Prevalence of self-reported psychiatric symptoms in relation to infection by *T. gondii *and *C. burnetii*

	***T. gondii***		***C. burnetii***	
	**Seropositive**	**Seronegative**	**Seropositive**	**Seronegative**
	**N = 166**	**N = 204**	**N = 130**	**N = 292**
*N (%)*				
*Fatigue*	27 (16.3)	33 (16.2)	18 (13.9)	43 (14.7)
*Concentration difficulties*	9 (5.4)	13 (6.4)	3 (2.3)	19 (6.5)
*Depression*	7 (4.2)	11 (5.4)	3 (2.3)	15 (5.1)
*Depressive ideas*	10 (6.0)	13 (6.4)	5 (3.9)	18 (6.2)
*Psychiatric morbidity*	10 (6.0)	15 (7.4)	6 (4.6)	19 (6.5)

**Table 2:** Odds ratios for self-reported psychiatric symptoms in relation to *T. gondii *and *C. burnetii*

	***T. gondii***	***C. burnetii***
	**OR (95% CI)**	**OR (95% CI)**
*Fatigue (n = 62)*		
Seronegative	1.00	1.00
Seropositive	1.18 (0.66–2.14)	0.92 (0.52–1.64)
*Concentration difficulties (n = 22)*		
Seronegative	1.00	1.00
Seropositive	0.97 (0.41–2.28)	0.32 (0.10–1.06)
*Depression (n = 18)*		
Seronegative	1.00	1.00
Seropositive	0.81 (0.29–2.30)	0.47 (0.13–1.67)
*Depressive ideas (n = 23)*		
Seronegative	1.00	1.00
Seropositive	1.11 (0.44–2.77)	0.62 (0.23–1.69)
*Psychiatric morbidity(n = 25)*		
Seronegative	1.00	1.00
Seropositive	0.88 (0.38–2.04)	0.71 (0.28–1.84)

Odds ratios account for sample clustering by farm holding

Odds ratios were adjusted for sex and age in 10-year bands (<30, 30–39, 40–49, 50–59, 60–69, 70+)

## Discussion

This study does not provide evidence to support the hypothesis that infection by *Toxoplasma gondii *is associated with difficulties in concentration. Given the relatively common exposure to latent toxplasmosis in both the general population and in certain occupational cohorts, even a relatively weak association with neuropsychiatric outcome might be of potential public health interest. However, no strong evidence to support such an association has been reported to date.

Havlicek and colleagues [1] reported significantly longer reaction times for completion of a computerised version of a psychomotor test amongst 60 *Toxoplasma*-positive individuals compared to 56 *Toxoplasma*-negative individuals, although the difference in reaction time was only up to 17 miliseconds. The authors interpreted this possible behavioural change as a manipulation activity to promote transmission of the parasite; delayed reaction times in rodents could increase the chance of transmission into a definitive host such as the cat. Flegr and collagues [2] investigated this hypothesis further by comparing the seroprevalence of latent toxoplasmosis in 146 subjects involved in traffic accidents (assumed to have delayed reaction) and 446 members of the general public in the same geographical area. Subjects with latent toxoplasmosis were 2.65 times more likely (95% CI 1.76–4.01) to be identified as having a traffic accident than those who were seronegative. However the study was limited by the sampling of both cases and controls relying on availability of archived blood samples for serological testing of toxoplasmosis thus increasing the possibility of selection bias, together with inadequate consideration of possible confounders.

Furthermore there is no evidence from this study to support the hypothesis that infection by *Coxiella burnetii *is associated with symptoms of fatigue. Wildman and colleagues [5] have previously reported that symptoms of fatigue were more common in 77 individuals assessed ten years after exposure to Q fever in a UK outbreak than in matched unexposed controls (65% vs 35% P < 0.001). Their study benefited from a comprehensive assessment of fatigue, a matched design to control for age, sex and smoking status, and a relatively high response rate amongst those exposed to Q fever (84%). However only 36% of the matched controls participated in the study, suggesting a possible selection bias. Finally due to the nature of self-report questionnaires and knowledge of study hypotheses the possibility of response bias could not be ruled out.

### Strengths and limitations

Exposure to both *T. gondii *and *C. burnetii *was common in this occupational cohort of farmworkers which is not surprising. Indeed the exposure to toxoplasma is common in the UK population as a whole (40% to 50%)[7], whereas occupational exposure is more important in the epidemiology of Q fever, with prevalence amongst farmworkers being two to three times higher than a comparison group of ambulance and police workers[8].

This survey benefited from using the CIS-R as a standardised assessment suitable for lay interviewers in assessing minor psychiatric disorder in an occupational setting. The computerised CIS-R assessment provides an easy, quick, inexpensive yet thorough assessment which is acceptable to interviewees and also helps to eliminate observer bias.

The prevalence of psychiatric symptoms was lower than expected in this cohort of farm workers[10], indicating that this is a relatively healthy occupational cohort with only very few individuals reporting clinically relevant levels of symptoms. Therefore unfortunately the statistical analyses are limited in power to test the study hypotheses. Although the response rate for the mental health survey was relatively high, it is possible that those subjects previously exposed to *T. gondii *or *C. burnetii *and who subsequently developed symptoms might have been more likely to drop out of the cohort either before recruitment or before this more recent survey ('healthy worker effect'). If this were the case we might underestimate the strength of association between exposure and psychiatric outcome. Finally we have no indicator of duration of infection for those individuals who are currently seropositive for either infectious agent which might affect the observed association if a long lag time is required between exposure and presentation of symptoms.

## Conclusions

We do not provide any evidence that infection with *Toxoplasma gondii *or *Coxiella burnetii *is associated with neuropsychiatric morbidity, in particular with symptoms of poor concentration or fatigue.

## List of abbreviations

*T. gondii *– *Toxoplasma gondii*

C. burnetii – Coxiella burnetii

CIS-R – Revised Clinical Interview Schedule

OR – odds ratio

95% CI – ninety five percent confidence interval

## Competing interests

The authors declare that they have no competing interests.

## Authors' contributions

HT completed the statistical analyses and drafted the manuscript, DT and RS conceived of the PHLS Farm Cohort study and participated in its design and coordination, GL developed the psychiatric assessments and advised in their use, AS advised on the study hypotheses under investigation. All authors read and approved the final manuscript.

## Pre-publication history

The pre-publication history for this paper can be accessed here:


